# Rural Social Participation through Osekkai during the COVID-19 Pandemic

**DOI:** 10.3390/ijerph18115924

**Published:** 2021-05-31

**Authors:** Ryuichi Ohta, Akiko Yata, Yuki Arakawa, Koichi Maiguma, Chiaki Sano

**Affiliations:** 1 Community Care, Unnan City Hospital, 96-1 Iida, Daito-cho, Unnan 699-1221, Shimane Prefecture, Japan; 2 Community Nurse Company, Unnan City, 422 Satokata, Kisuki-cho, Unnan 699-1311, Shimane Prefecture, Japan; yataakiko0425@gmail.com; 3 Doctoral Program, Graduate School of Medicine, School of Social Medicine, The University of Tokyo, 17 Chome-3-1 Hongo, Bunkyo City, Tokyo 113-8654, Japan; addp1086@gmail.com; 4 Department of Law and Economics, Faculty of Law and Literature, Shimane University, 1060 Nishikawatsu cho, Matsue 690-8504, Shimane Prefecture, Japan; zzkuma@soc.shimane-u.ac.jp; 5 Department of Community Medicine Management, Faculty of Medicine, Shimane University, 89-1 Enya cho, Izumo 693-8501, Shimane Prefecture, Japan; sanochi@med.shimane-u.ac.jp

**Keywords:** community activity, COVID-19, health promotion, loneliness, Osekkai, social good, social participation

## Abstract

We investigated the effects of enabling Osekkai, the traditional Japanese behavior of creating a helping culture, on social participation among rural people in rebuilding social connections that can be vital during the coronavirus diseases 2019 (COVID-19) pandemic. The subjects of this cross-sectional study were people interested in the Osekkai conference (control group) and those actively involved in Osekkai activities (exposure group). The primary outcome of social participation was measured as the frequency of meeting and the number of friends or acquaintances. The demographic data of the participants and process outcomes were measured using a questionnaire provided to all 287 registered participants. The effective response rate was 64.5% (185 responses). The involvement in Osekkai conferences was statistically associated with a high frequency and number of meetings with friends or acquaintances (*p* < 0.001 and 0.048, respectively). A health check was significantly associated with the number of friends or acquaintances met in the previous month, while high social support was significantly associated with loneliness. Thus, we confirm that Osekkai contributes to high social participation, although we see no relationship with loneliness. Future studies should investigate this cause-and-effect relationship and promote culturally sensitive activities to improve social and health outcomes in rural Japan.

## 1. Introduction

Losing social connections can upset rural communities and place their sustainability at risk. The result of such a loss is less social participation and eventually maladaptive behaviors such as harmful habits or serious health issues such as depression and cardiovascular diseases. The loss of community thus affects the quality of life (QOL) [1,2,3]. Issues of privacy, employment, and cultural diversity have weakened social connections in rural areas [4]. Rural residents less frequently share their private information and help each other now than in the past [5]. The migration of long-time rural residents as well as the influx of new people [5,6] have especially increased loneliness, especially among the elderly [3,7,8]. Aggravating this scenario is the spread of the novel coronavirus disease 2019 (COVID-19). The forced home isolation among rural residents has further reduced social connections in communities [9,10,11]. It is considered pertinent to understand how COVID-19 has reduced social participation and, thus, connections in communities [12].

To rebuild social connections, culturally sensitive activities should be promoted. These are activities that are best understood within a specific culture and involve indigenous people. They can help build relationships and increase social participation [13,14,15]. Osekkai is one such cultural practice. It is a traditional Japanese behavior wherein people help others to create a safe and comfortable community, and it can increase social participation among its practitioners [16]. Osekkai is an unconscious behavior in Japanese communities. However, aging and multicultural societies see fewer acts of Osekkai, which can harm relationships in communities. Due to the coronavirus diseases 2019 (COVID-19) pandemic, opportunities for social participation have decreased, and so has the presence of Osekkai. Revitalizing this practice can help rebuild social connections in rural communities. The countermeasures to contain COVID-19 have forced governments to suppress rural social norms such as Osekkai. The lack of social interaction and participation can deteriorate people’s mental and physical health [9,10,11]. Thus, we must find ways to revitalize culturally sensitive activities such as Osekkai under the constraints of COVID-19 for the improvement of people’s health conditions.

Japanese rural communities regularly held Osekkai conferences to revitalize this practice during the pandemic. It led to the forming of new relationships in communities and improved the number of social interactions and participation of those involved. Governments and companies steered various interventions targeting improvement in peoples’ social participation using information and communication technology during the COVID-19 lockdown, which may lead to its improvement. A literature review shows a lack of scientific research regarding interventions driven by community members and local governments. No study has clearly shown the relationship between culturally sensitive activities and higher social participation among practitioners. Thus, our research question is, Is there a relationship between involvement in Osekkai and social participation in rural areas? Clarifying the effects of such Osekkai conferences can motivate other rural communities to follow the trend and improve their community’s conditions, especially given the decreasing global social interactions due to COVID-19, causing multiple health problems [17,18]. We thus investigated the effects of promoting Osekkai on rural social participation in rebuilding social connections.

## 2. Materials and Methods

In this study, we used a cross-sectional design. First, we assessed the quality of the intervention of the Osekkai conference based on the Reach, Efficiency/Effectiveness, Adoption, Implementation, and Maintenance (RE-AIM) framework [19]. *Reach* clarifies how one reaches out to those who need a specific intervention; *efficacy/effectiveness* gauges how well the intervention is working; *adoption* clarifies how to design for dissemination and develop organizational support to deliver the intervention; *implementation* deals with ensuring the intervention is feasible and delivered properly; and *maintenance* relates to ensuring long-term benefits and institutionalization of the intervention and continued community capacity.

To assess reach, we calculated the number of participants in the conference; to assess efficacy and effectiveness, we measured the participants’ social participation in the frequency of meeting friends or acquaintances and the number of friends or acquaintances; to assess adoption, we measured the number and location of conferences; to assess implementation, we measured the process of each conference based on the criteria of the conference protocol; to assess maintenance, we measured people’s continued involvement in the conferences and their effect on Osekkai. The study duration was from September 2019 to March 2021.

### 2.1. Setting

Unnan City, located in the southeast of Shimane Prefecture, is one of the most rural areas in Japan. In 2020, the total population of Unnan was 37,638 (18,145 males and 19,492 females), with 39% being over 65 years old. This proportion is expected to reach 50% by 2025 [20]. The situation of the city was typical of Japanese rural settings. There are 30 autonomous community organizations in Unnan City, each of which has various functions for managing social issues such as social isolation, accessibility to medical care, and succession of traditional activities. Each district has at least one autonomous community organization.

### 2.2. Osekkai Conferences

Emergent situations require voluntary activities for communities wherein human resources are limited and isolated people are not able to seek help. In Japan, both formal and informal rural care providers establish flexible meetings called Osekkai conferences, which allow people to get together and discuss community problems. Conferences currently take place in small groups to avoid the spread of COVID-19 in rural communities [16]. At the Osekkai conferences, rural residents present their community’s problems and social issues. The conferences involve multiple people with different professional backgrounds, such as medicine, law, public health, rehabilitation, architecture, community development, and transportation. Conference attendees collaborate to solve community problems by sharing their experiences and suggesting solutions to similar problems. Finally, an Osekkai plan is established, which is carried out in each rural community using the suggested resources. The results of the plan’s provision are then shared in the following Osekkai conferences. Through continual discussions, Osekkai plans can be revised to improve the quality of care. Such routine discussions can foster new, effective collaborations between providers of different resources. The conference that is the focus of our attention began in September 2020 and mainly took place in Kisuki, Unnan City.

### 2.3. Participants

The participants included anyone interested in the Osekkai conference. The conference information was provided by the Unnan City Hall via social media, and the city’s local newspapers and interested participants called upon or sent a mail to the office of the conference to register. The Osekkai conference had five types of activities: consulting on difficulties in communities with conference staff and planning, performing, supporting, and accepting Osekkai. The participants gathered at the community center where the conference was held on the day of the conference. During March 2021, a questionnaire was provided to them online or in letter form, based on their preferences. The responses were collected in April 2021.

### 2.4. Measurements

The exposure group included participants involved in activities of the Osekkai conference, and the control group included participants who were motivated but did not participate in the activities. As a primary outcome of social participation, the frequency of meeting friends or acquaintances (more than 4 times/week, 2–3 times/week, 1 time/week, 1–3 times/month, several times/year, no meeting) and the number of friends or acquaintances met in the previous month (no meeting, 1–2 persons, 3–5 persons, 6–9 persons, more than 10 persons) were measured [21]. As secondary outcomes, the degree of loneliness was based on the Japanese version of the three-item UCLA Loneliness Scale among community-dwelling older adults (score range: 3–9) [22]. We also measured their perception of changing the frequency of meeting with friends and acquaintances and the time spent in sharing joy with others (increased enough, relatively increased, no change, relatively decreased, and strongly decreased). The number of participants, content, and adherence to the standards of each conference were calculated at the time of each conference. We also collected additional data such as involvement in the five activities of Osekkai conferences, age, gender, residence (Unnan City or outside), the presence of social support (yes or no) [20], whether participants received regular health checks (yes or no), tobacco use (*yes* or *no*), habitual consumption of alcohol (*yes* or *no*), educational background (*elementary school*, *junior high school*, *high school*, or *university or higher*), and socioeconomic status (*rich*, *relatively rich*, *not poor*, *relatively poor*, or *poor*).

### 2.5. Analysis

Based on the test of normality, Student’s *t*-test was performed on parametric data and the Mann–Whitney U-test was performed on nonparametric data. The participants were categorized into two groups, exposure or control, depending on their involvement in conference activities (exposure = consulting on difficulties in communities to the conference staff; organizing the conference; and planning, performing, supporting, or accepting Osekkai; control = not). The variables were categorized binomially: sex (male = 1, female = 0), tobacco use (yes = 1, no = 0), habitual consumption of alcohol (yes = 1, no = 0), educational level (above high school =1, no = 0), social support (have or relatively have = 1, relatively do not have or do not have = 0), socioeconomical state (high (rich, relatively rich) = 1, low (relatively poor or poor) = 0), perception of changing the frequency of meeting with friends and acquaintances and time spent in sharing joy with others (increased enough or relatively increased = 1, no change, relatively decreased or strongly decreased = 0), frequency of meeting friends or acquaintances (more than 4 times/week or 2–3 times/week or 1 time/week = 1, 1–3 times/month or several times/year or no meeting = 0), and the number of friends or acquaintances met in the previous month (more than 10 persons or 6–9 persons = 1, 3–5 persons or 1–2 persons or no meeting = 0). Multivariate logistic regression analysis was performed to assess whether involvement in the conference activities was associated with all the related factors with differences between the exposure and control groups. Cases with missing data were excluded from the analysis. Statistical significance was set at *p* < 0.05.

### 2.6. Ethical Consideration

The anonymity and confidentiality of patient information was ensured throughout the study. Only anonymous data were provided by the Unnan Public Health Center. The research information was posted on the hospital website without including any patient information. The contact information of the hospital representative was likewise listed on the website so that questions about the research could be answered at any time. All procedures included in this study were performed in compliance with the Declaration of Helsinki and its subsequent amendments. The Unnan City Hospital Clinical Ethics Committee approved the study protocol (Nno. 20190021).

## 3. Results

### 3.1. Osekkai Conference Provisions

**Figure 1:**
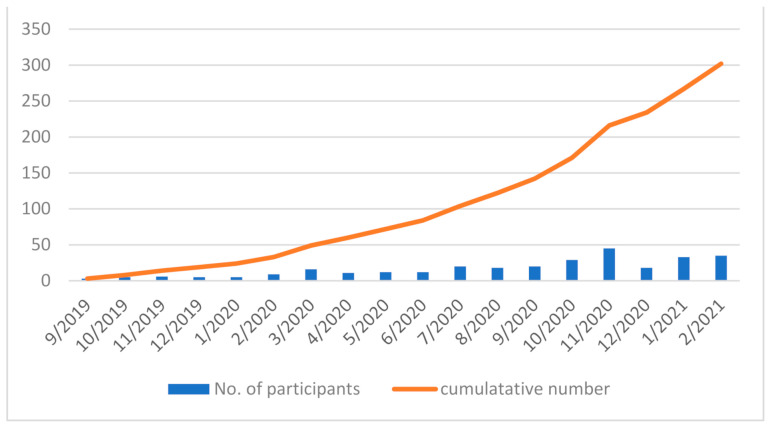
Participants’ trend in each conference and cumulative numbers.

During the study period, 19 conferences were held and 302 participants participated in the conferences. The average number of participants was 16.8 (standard deviation (SD) = 11.9) (Figure 1). Each conference was facilitated by the organizers, and the average number of organizers in each conference was 5.3 (SD = 0.89). All the conferences were held as per schedule, and three Osekkai topics were discussed among the participants.

### 3.2. Demographics of the Participants

The effective response rate to the questionnaire provided to all 287 registered people was 64.5% (185 responses). The average age of the participants in the exposure and control groups was 44.34 (SD = 16.59) and 42.50 (SD = 19.75) years, respectively. Female participants dominated both groups. The exposure group had a statistically lower rate of living with the family than the control group. The exposure group showed increasing frequency of meeting friends and timings of sharing joy with statistical significance compared with the control group (*p* < 0.001). There were no statistical differences between the two groups regarding education, socioeconomic conditions, habitual consumption of alcohol, tobacco use, regular health checks, medical care, residence, and social support (Table 1).

### 3.3. Relationship between Osekkai Participation and Social Participation

In the comparison of social participation between the two groups, involvement in Osekkai conferences was significantly associated with a high frequency and a greater number of meetings with friends or acquaintances (*p* < 0.001 and 0.048, respectively). Having a health check was significantly associated with the number of friends or acquaintances met in the previous month, while high social support was significantly associated with loneliness (Table 2).

## 4. Discussion

This study clarified that the involvement in culturally sensitive activity such as Osekkai can be associated with social participation and especially foster relationships with friends or acquaintances during the pandemic. We found no clear association between Osekkai involvement and loneliness. The cause-and-effect relationship of Osekkai with social participation and loneliness should be further studied in the future. For Osekkai conferences to truly succeed, their provision should be distributed more effectively. This will further ease loneliness among participants during the pandemic.

Health interventions should be based on the framework of previous effective interventions. We find that the Osekkai conferences also followed conventional protocols based on the RE-AIM framework [19]. Our intervention at the Osekkai conferences helped increase the number of participants and adherence to its guidelines [19]. However, its duration of about one year is insufficient to significantly change social and health conditions [23]. Further, the conference provisions were revised to suit the context of the COVID-19 pandemic and then assessed for effectiveness and efficiency based on various community aspects. In the current pandemic, the situation of the spreading infection is continuously changing because of the appearance of new variants and additional waves of COVID-19 [24]. The continued provision of culturally sensitive activities requires consideration of the pandemic conditions and the revision of concrete provisions. We encourage similar culturally sensitive activities to rebuild social relationships around the world by adapting them to the dynamic pandemic situations [25].

Importantly, our study shows a significant relationship between involvement in Osekkai conferences and the number and frequency of meeting with friends or acquaintances. The literature also confirms that community activities (e.g., recreational activities or exercise programs) under central or local government initiatives can improve social participation [26,27,28]. The Osekkai conferences are one such avenue for rural residents to actively regain their sense of community. The conferences are driven by community workers and locals striving to resolve problems and difficulties in their community. Such difficulty-driven activities can motivate participants and stimulate their spirit as volunteers [29,30,31]. Thus, Osekkai conferences can drive social participation among motivated community members as well those people looking forward to resolve problems. Given our finding of positive association, subsequent studies should investigate the relationship in longitudinal study designs to clarify the cause-and-effect model.

Interestingly, we found no relationship between loneliness and Osekkai. We explain this result as follows: First, improving health and social conditions requires continuous and more intervention [32,33]. Despite the routine conferences, pandemic restrictions made it difficult to fully realize Osekkai. While pandemic-related issues can inspire residents to participate in conferences, the uncertainty of the COVID-19 outbreak itself can mean that circumstances change unexpectedly [12,34]. As a result, the actions of Osekkai might be revised quickly or canceled, thus reducing actual activities. Therefore, Osekkai activities might not address loneliness. However, we also found that a significant association between social support and loneliness, implying that people experiencing loneliness perceive less social support from society. As studies have suggested, more multifactorial interventions can solve loneliness [32,35], although, from a global perspective, cultural differences can complicate the causes of and solutions for loneliness [36,37]. People with loneliness need both social support as well as meaningful roles to fulfill, to manage their lives [38,39]. We further found that participants who regularly underwent health checkups tended to have lower social participations with respect to meeting others, which could be a result of health consciousness regarding the risk of COVID-19. Health-conscious people can be sensitive to the COVID-19 pandemic and have their own home-based health control methods, which may lead to their reduced social participation [40,41].

We believe that involvement in Osekkai can initiate new relationships among its participants, who can then build their roles in communities. The conferences can support participants in this process by involving all community stakeholders [30,31]. Through improved content, culturally sensitive activities can address loneliness during the pandemic. COVID-19 also presents a new context within which to study the association between culturally sensitive activities and social participation. The COVID-19 pandemic can impinge on social interaction in rural communities because of the lack of healthcare resources and the same standard of limitation of social interaction based on urban areas. Rural governments and social community members should be motivated to progress their own culturally sensitive activities that can be understood by indigenous people for rebuilding social relationships and improving social participations, which can contribute to health promotion of the people.

Our study is limited by its cross-sectional study design, which cannot show the cause-and-effect relationship. We recommend longitudinal studies to overcome this problem. Since we conducted our study in rural Japanese communities, where people were already motivated to engage in culturally sensitive activities, our sample has selection bias. Therefore, by investigating a broader range of communities and including people who have never been involved in Osekkai conferences can offer a more nuanced understanding. Moreover, participants’ motivation for partaking in the Osekkai conferences, such as being invited by their relatives, friends, or families, can be an important factor and should, therefore, be examined in future studies. Further, Japan is an aging country, and many developing and developed economies will soon follow this trend as well. Our findings can be helpful for such potential communities. Finally, our sample size was relatively small. A larger sample size with a wider range of demographics can address this limitation.

## 5. Conclusions

This study clarified that the culturally sensitive activity of Osekkai can be associated with social participation and especially foster relationships with friends or acquaintances during the pandemic. The cause-and-effect relationship of Osekkai with social participation and loneliness should be further studied in the future. For Osekkai conferences to truly succeed, their provision should be distributed effectively.

## Figures and Tables

**Table 1:** Demographics and perception of participation in Osekkai among the participants.

	Participation in Osekkai		
Factor	Exposure	Control	*p*-Value
N	119	66	
Age, men, mean (SD)	44.34 (16.59)	42.50 (19.75)	0.5
Sex (%)			
Male	76 (63.9)	43 (65.2)	0.874
Female	43 (36.1)	23 (34.8)	
Education (%)			
Low	5 (4.2)	1 (1.6)	0.665
High	113 (95.8)	60 (98.4)	
Socioeconomic status (%)			
Low	80 (67.2)	50 (76.9)	0.18
High	39 (32.8)	15 (23.1)	
Habitual alcohol use (%)	62 (52.5)	28 (43.1)	0.28
Tobacco use (%)	7 (6.0)	4 (6.2)	1
Regular health check (%)	73 (61.9)	44 (68.8)	0.419
Living with family (%)	93 (78.2)	61 (93.8)	0.006
Living location (%)			
Outside	32 (27.1)	14 (21.5)	0.478
Unnan City	86 (72.9)	51 (78.5)	
Social support (%)			
High	105 (89.0)	56 (87.5)	0.81
Low	13 (11.0)	8 (12.5)	
Increasing frequency of meeting friends (%)			
Yes	64 (53.8)	10 (15.2)	<0.001
No	55 (46.2)	56 (84.8)	
Increasing timings of sharing joy (%)			
Yes	56 (47.1)	7 (10.6)	<0.001
No	63 (52.9)	59 (89.4)	

**Table 2:** Relationship of Osekkai participation with social participation and loneliness.

	Frequency of Meeting			Number of Meetings			Loneliness		
Factor	AOR	95% CI	*p*-Value	AOR	95% CI	*p*-Value	AOR	95% CI	*p*-Value
Osekkai	3.64	1.67–7.93	<0.001	2.05	1.01–4.17	0.048	0.76	0.37–1.55	0.44
Age	0.98	0.96–1.01	0.067	1.02	1.00–1.05	0.13	0.99	0.97–1.02	0.28
Male	0.73	0.34–1.56	0.42	0.61	0.30–1.26	0.18	1.2	0.58–2.50	0.62
Higher education	3.28	0.34–31.81	0.31	0.14	0.01–1.45	0.098	1.96	0.26–15.01	0.52
High SES	0.59	0.27–1.31	0.19	0.75	0.35–1.63	0.47	1.07	0.50–2.33	0.86
Living with family	1.48	0.59–3.68	0.4	0.88	0.35–2.23	0.79	0.42	0.16–1.08	0.07
Residence	1.11	0.49–2.54	0.81	0.52	0.23–1.19	0.12	0.86	0.38–1.98	0.72
High SS	1.88	0.57–6.16	0.3	0.57	0.18–1.87	0.35	17.2	1.98–150.01	<0.001
Habitual alcohol use	1.31	0.65–2.66	0.45	0.63	0.32–1.26	0.19	0.93	0.47–1.85	0.83
Health check	1.7	0.79–3.70	0.18	0.41	0.19–0.88	0.021	1.49	0.70–3.16	0.3

Frequency of meetings: the frequency of meeting friends or acquaintances; number of meetings: the number of friends or acquaintances met in the previous month; AOR: adjusted odds ratio; CI: confidence interval; SES: socioeconomic status; SS: social support; Osekkai: involvement in activities of Osekkai conferences.

## Data Availability

The datasets used and/or analyzed during the current study may be obtained from the corresponding author upon reasonable request.
